# Soil function reshaping and crop yield driving mechanisms in saline-alkali soil under freezing saline water irrigation

**DOI:** 10.3389/fpls.2026.1856608

**Published:** 2026-06-08

**Authors:** Junjie Li, Zhongyi Qu, Liping Wang, Pengcheng Tang, XiaoYu Gao, Gerile Hasi, Dongliang Zhang

**Affiliations:** 1 State Key Laboratory of Water Engineering Ecology and Environment in Arid Area, Inner Mongolia Agricultural University, Hohhot, China; 2 College of Water Conservation and Civil Engineering, Inner Mongolia Agricultural University, Hohhot, China; 3 College of Energy and Environment, Inner Mongolia University of Science and Technology, Baotou, China; 4 Yinshanbeilu Grassland Eco-hydrology National Observation and Research Station, China Institute of Water Resources and Hydropower Research, Beijing, China

**Keywords:** crop growth, freezing saline water irrigation, multifunctionality, saline soils, soil thresholds

## Abstract

**Introduction:**

Freshwater scarcity and soil salinization constrain agricultural production in arid and semi-arid regions. Freezing saline water irrigation (FSWI) offers a potential strategy for using saline water during the freeze-thaw period, but the optimal irrigation amount and its yield-driving mechanisms remain unclear.

**Methods:**

Here, a three-year field experiment was conducted from 2022 to 2024 in Dalad Banner, Inner Mongolia, China, with four FSWI treatments of 0, 90, 180 and 270 mm. We evaluated pre-sowing soil hydrothermal, salinity and microbial functional indicators, and assessed sunflower growth, yield and seed quality under uniform agronomic management.

**Results:**

Among the treatments, FSWI180 showed the best overall performance. It maintained favorable pre-sowing soil moisture and salinity conditions, increased yield by 93% relative to the non-irrigated control, and improved seed oil content to 43.96%. It also achieved the highest comprehensive growth and quality indices, with mean GI and QI values of 2.126 and 1.182, respectively. XGBoost-SHAP analysis identified soil moisture, total salinity, electrical conductivity and community robustness as the main yield-driving factors, together accounting for 74.75% of the total contribution.

**Discussion:**

These results indicate that 180 mm FSWI can coordinate soil water storage, salinity regulation and microbial functional stability, thereby supporting stable yield improvement and high-quality sunflower production in cold arid saline-alkali regions.

## 1 Introduction

Agriculture in arid and semi-arid regions has long been constrained by the dual challenges of freshwater scarcity and soil salinization (Qin et al., 2025). Insufficient freshwater reduces the reliability of irrigation, whereas salt accumulation diminishes soil productivity and restricts stable and high crop yields (Cheng et al., 2021; Du et al., 2023). As competition for agricultural water continues to intensify, saline water, as an important unconventional water resource, has gradually become a viable option for alleviating freshwater shortages (Liu et al., 2024). However, the use of saline water also entails risks. Long term or high intensity saline irrigation during the growing season may lead to salt accumulation in the root zone, soil sodification and structural degradation, thereby increasing the risks to agricultural production (Yin et al., 2021). Recent studies on long term saline irrigation have shown that increases in irrigation water salinity can significantly elevate soil electrical conductivity and sodium adsorption ratio, while also adversely affecting soil pore characteristics and enzyme activity (Zhang et al., 2024a; Ye et al., 2025). These findings suggest that the effective use of saline water depends not only on water quality itself, but also on irrigation timing and irrigation method (Xin et al., 2024).

Unlike direct saline irrigation during the growing season, FSWI is a winter irrigation based salt leaching approach in which the critical period of action occurs during the pre-sowing freeze thaw stage. Its effects are not limited to replenishing soil water, but may also alter water and salt migration, thermal conditions and structural properties through the processes of freezing and thawing (Guo et al., 2021; Li et al., 2026b). Previous studies have shown that saline ice meltwater exhibits pronounced water salt separation characteristics during infiltration. The initial meltwater often has a relatively high salt concentration, whereas subsequent meltwater may display lower salinity and a lower sodium adsorption ratio, thereby promoting the redistribution of water and salts in saline soils (Guo and Liu, 2015; Xiao et al., 2017). Soil column experiments have further confirmed that infiltration of saline ice meltwater can increase soil moisture content and influence salt migration within the soil profile (Yang et al., 2021; Zhang et al., 2023). Field studies have likewise demonstrated that FSWI can regulate soil water, heat and salt dynamics. Appropriate irrigation amounts can facilitate water storage and salt leaching, whereas excessive irrigation may induce a risk of secondary salinization (Li et al., 2025; Han et al., 2025). Current research on FSWI has mainly focused on soil salt migration, soil moisture and crop responses. Although substantial progress has been made, existing studies have largely centered on single indicators such as soil moisture content, electrical conductivity or final yield, and a systematic understanding of pre sowing soil functional change under FSWI remains lacking (Qiao et al., 2026; Li et al., 2026a). In particular, few studies have incorporated soil water and salt functions, soil microbial functions, and soil temperature and structural functions into a unified analytical framework. There is also a lack of in depth explanation of how improvements in the pre sowing soil environment generate persistent legacy effects during the subsequent growing season.

The regulatory effects of FSWI are inherently multidimensional and complex. Its outcomes are reflected not only in changes in pre-sowing soil function, but also in crop growth, yield formation and grain quality (Li et al., 2023; Luo et al., 2025; He et al., 2025). Different treatments may perform well for some indicators, but not necessarily for others. Therefore, reliance on a single indicator or simple treatment comparisons is insufficient for accurately evaluating the overall effects of different irrigation amounts (Liu J. et al., 2025; Lin et al., 2025). Principal component analysis can integrate information from multiple indicators and provide a basis for comprehensive optimization among treatments (Wang S. et al., 2025). At the same time, crop yield is usually co-regulated by multiple soil factors and crop traits, and these relationships are often nonlinear (Zhang et al., 2022). Recent studies on FSWI have already shown that soil moisture and salinity are important determinants of crop growth, and that excessive irrigation can alter both the strength and direction of their effects (Guo et al., 2024). Against this background, conventional significance testing alone is insufficient to determine dominant variables, effect directions and favorable ranges for yield improvement (Li J. et al., 2024; Ma et al., 2025). Compared with traditional machine learning methods such as random forest, XGBoost is better suited to this study because its gradient boosting and regularization mechanisms improve the ability to capture complex nonlinear interactions among soil water, salinity, structure, temperature and microbial indicators, while reducing overfitting risk. Moreover, SHAP overcomes the limited interpretability of black-box models by quantifying the relative contribution, positive or negative effect direction, and threshold intervals of each soil functional variable. Therefore, combining principal component analysis with XGBoost-SHAP enables a continuous analytical pathway from comprehensive treatment evaluation, to identification of the mechanisms underlying yield formation, and further to extraction of key regulatory intervals. This also reflects the novelty of the present study in both its analytical framework and methodological approach (Jones et al., 2022; Aksoy et al., 2024).

Based on this rationale, we established FSWI treatments with different irrigation amounts and maintained consistent agronomic management during the growing season to isolate the legacy effects of the pre-sowing soil environment on subsequent crop performance. Specifically, this study aimed to: (1) systematically evaluate how different FSWI amounts regulate pre-sowing soil multifunctionality, including soil water-salt, thermal-structural and microbial functions during the freeze-thaw period; (2) determine how these changes affect sunflower growth, yield formation and seed quality during the growing season; and (3) identify the dominant soil functional drivers and threshold intervals controlling yield formation. Compared with previous studies that mainly focused on individual soil water-salt indicators or final crop yield, this study integrates pre-sowing soil multifunctionality with crop performance evaluation and combines PCA with XGBoost-SHAP to link comprehensive treatment assessment, yield-driving mechanism identification and key threshold extraction. These analyses provide a theoretical basis and practical reference for the safe use of saline water resources, irrigation amount optimization and agricultural water management in cold arid saline-alkali land.

## 2 Materials and methods

### 2.1 Description of the study area

**Figure 1:**
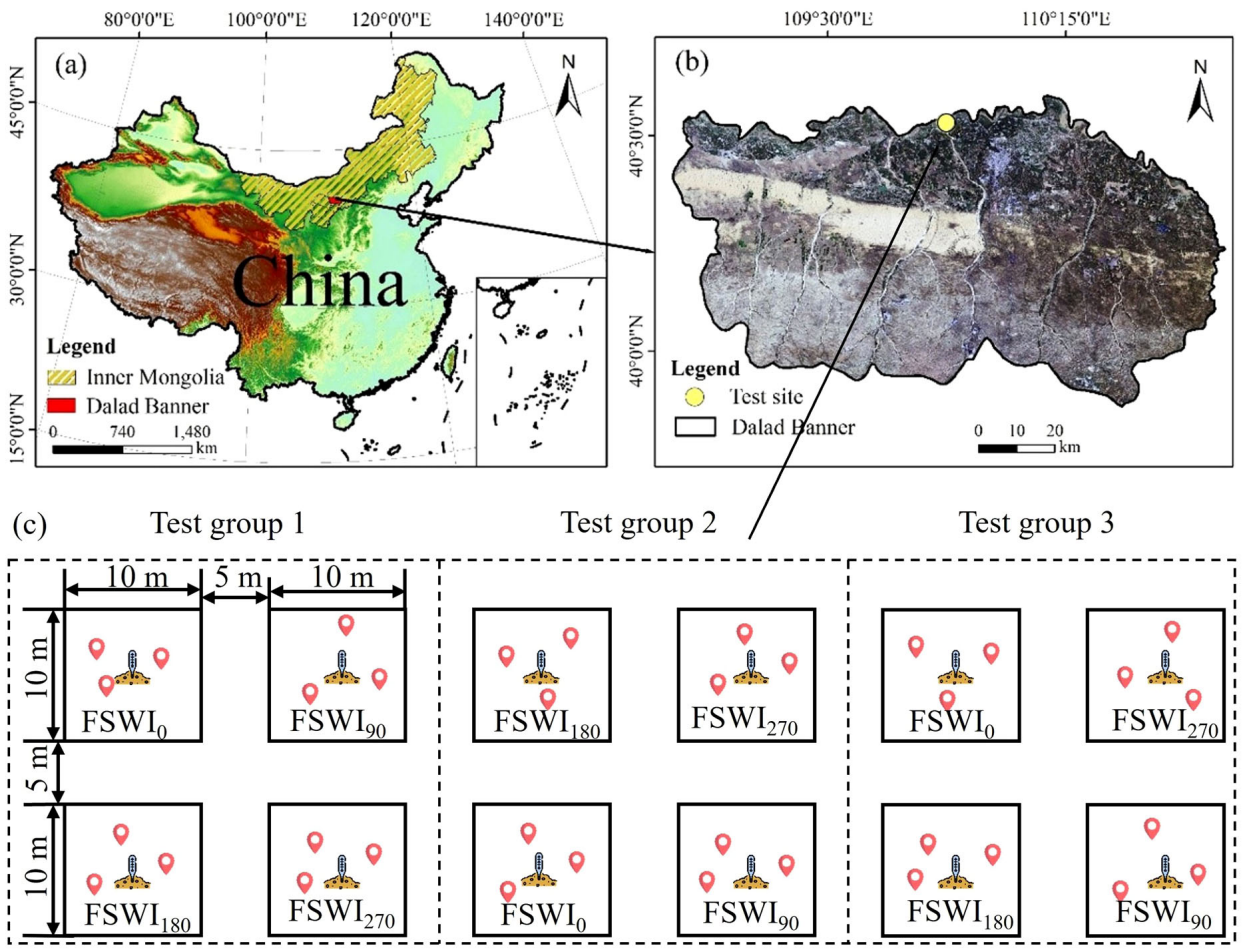
Location of Inner Mongolia **(a)**, Darat Banner **(b)**, layout of the test area **(c)**. The red icons in the figure indicate the positions of the sampling points.

**Figure 2:**
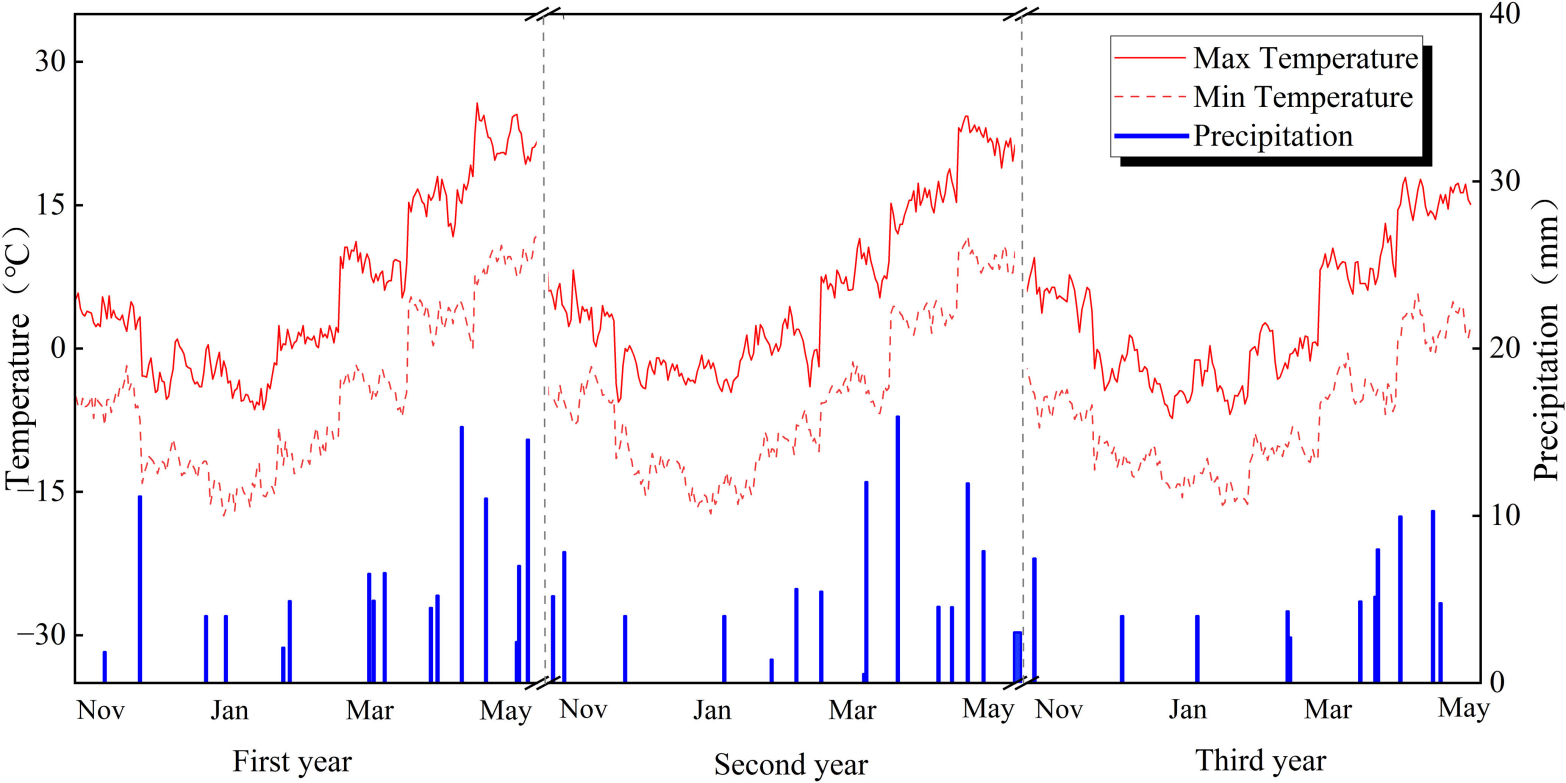
The temperature data during the three-year trial period include the highest temperature, the lowest temperature and the precipitation amount.

The experiment was conducted from October 2022–2024 at the Dalate Banner, Ordos (**Figure 1b**), China (**Figure 1a**) test site (109°87’E, 40°49’N, 1004 MSL). This arid region is in the temperate continental climate zone, low rainfall, exhibits cold winters and hot summers, and presents large temperature differences between day and night. The average annual sunshine is 3000 h, average annual temperature is 6.5 °C, frost-free period is 140 d, average annual evaporation is 2200 mm, and average rainfall is 300 mm (**Figure 2**). The average soil salinity in the 0–40 cm soil layer of the test area was 10.23 g kg^-1^, and the average soil salinity in the 40–120 cm soil layer was 11.02 g kg^-1^. The pH values was 9.

### 2.2 Experimental design

To investigate the feasibility of utilizing freezing irrigation to achieve efficient environmental use of water resources, freezing irrigation field trials were conducted based on different saline water amounts. According to the results of an earlier study (Guo et al., 2014), the experiment was set up with four groups of treatments according to different irrigation levels: 90 mm (FSWI_90_), 180 mm (FSWI_180_), 270 mm (FSWI_270_), and control without irrigation (CK). Three replications were set up for each treatment, with a total of 12 experimental plots, each with an area of 100 m^2^ (10 m×10 m) (**Figure 1c**).

The plots were completely randomly arranged, and an isolation zone with a spacing of 5 m was set up in each plot to reduce the impact of lateral seepage between treatments. The irrigation water mineralization rate was 8.52 g L^-1^, and the pH was 9. When the average daily temperature was less than -5 °C, salty water in a cistern was extracted for icing irrigation. In the first round of irrigation, 30% of the entire amount of water was irrigated, and when this irrigation water was completely frozen, the remaining 70% was irrigated, the two rounds of irrigation lasted a total of three days. In the three study years, irrigation began on 15 November 2022, 18 November 2023, 16 November 2024, and the salty water from the irrigation froze at the top of the soil.

To avoid potential interference from differences in cultivation practices, sunflower field management was conducted using a unified conventional planting pattern across all treatments. The sunflower cultivar was Meikui 363. Seeds were sown by mechanical drilling with equal row spacing, with a row spacing of 40 cm and a plant spacing of 60 cm. Irrigation was applied using mulched drip irrigation, with Yellow River water as the irrigation source, and drip irrigation was conducted separately at the seedling, budding, flowering and grain-filling stages. A nitrogen-phosphorus-potassium compound fertilizer was applied, with mass fractions of N, P_2_O_5_ and K_2_O of 14%, 16% and 15%, respectively, at a total application rate of 450 kg ha^−1^. All planting operations were kept consistent within the experimental area and were completed by the same machinery team within two days, ensuring that there were no systematic differences in agronomic practices other than the target experimental factor.

### 2.3 Data collection and sampling methods

At the end of the freeze thaw period and before crop sowing, soil functional indicators in the 0 to 40 cm soil layer were measured to evaluate the regulatory effects of different amounts of FSWI on the basic pre sowing soil environment in saline alkaline land. According to their properties, the pre sowing soil functional indicators were classified into three categories, namely soil water and salt functions, soil temperature and structural functions, and soil microbial functions. All samples were collected according to a unified sampling protocol and were subsequently used to reveal changes in pre sowing soil function and to clarify the basis for their effects on subsequent crop performance.

#### 2.3.1 Soil water and salt

Soil water and salt functional indicators included soil water content (SWC), soil electrical conductivity (EC), total soil salinity (TSC), and sodium adsorption ratio (SAR). These indicators were mainly used to characterize the pre sowing soil water storage status, salinity level and sodification risk following FSWI, and thus represent the core variables for evaluating the pre sowing soil water and salt environment in saline alkaline land.

Soil water content (SWC) was determined by the oven-drying method. Fresh soil samples were collected, sealed immediately and transported to the laboratory. A homogenized subsample was dried at 105 °C to constant weight, cooled in a desiccator, and weighed to calculate SWC.

For the calculation of the sodium adsorption ratio (SAR), soil samples were air-dried, ground and passed through a 1 mm sieve. Soil extracts were then prepared, and the concentrations of soluble Na^+^, Ca²^+^ and Mg²^+^ were determined by atomic absorption spectrophotometry using a WYX-402C instrument (Shenyang, China). SAR was calculated from the concentrations of Na^+^, Ca²^+^ and Mg²^+^ in the extract using Equation 1:

(1) $$SAR=\frac{Na^{+}}{\sqrt{(Ca^{2+}+Mg^{2+})/2}}$$

where the concentrations of all ions are expressed in mmol L^−1^.

#### 2.3.2 Soil temperature and structure

Soil temperature and structural functional indicators included pore directness (PD), pore connectivity (PC), soil temperature (ST) and soil temperature gradient (STG). These indicators were mainly used to characterize the effects of FSWI on soil thermal status and physical structure during the freeze thaw process, thereby reflecting soil aeration, transport capacity and energy exchange conditions.

Soil temperature in the experimental area was continuously monitored using a multi-channel soil temperature sensor system (model TM 03, Hebei, China). Probes were installed by depth within the 0 to 40 cm soil layer to obtain temperature data, and the recording interval was set at 24 h. Each time soil cores were collected, the corresponding soil temperature data for the same period were simultaneously retrieved from the monitoring system. Soil temperature gradient was calculated using Equation 2.

(2) $$STG=\frac{ST_{1}-ST_{2}}{D}$$

where STG is the soil temperature gradient (°C cm^-1^), ST_1_ and ST_2_ are the soil temperatures measured at two adjacent soil depths, and D is the soil depth (cm).

Soil samples were imaged in three dimensions using an industrial computed tomography system (phoenix v|tome|x, GE, United States). Three replicates were prepared for each treatment, and cubic samples measuring 50 × 50 × 50 mm were scanned at an imaging resolution of 30 μm per pixel. During scanning, each sample was fixed on a rotating stage and rotated from 0° to 360° with an angular increment of 0.3° for full angle image acquisition. The X ray scanning parameters were set to a tube voltage of 180 kV, a tube current of 80 mA, an exposure time of 1250 ms for each projection, and a source to sample distance of 200 mm. After scanning, the acquired two dimensional tomographic slices were first reconstructed into three dimensional volumetric data, and image processing together with quantitative structural analysis was then carried out in Avizo software. Approximately 1,500 two dimensional slices were reconstructed for each sample and imported into Avizo. The continuous slices were first filtered to reduce background noise. The grayscale images were then segmented using a combination of threshold segmentation and the top hat method to accurately distinguish the pore phase from the solid particle phase. Based on the reconstructed three dimensional pore structure, connectivity analysis and pore segmentation were further performed to calculate pore structural parameters, including PD (mean pore diameter) and PC (connectivity).

#### 2.3.3 Microorganisms and enzyme activity

Soil microbial functional indicators included microbial diversity (MD), microbial community robustness (Rob), community stability (CS) and enzyme activity (EA). In this study, MD was used to represent the richness and diversity of soil microbial communities. Rob reflected the ability of the microbial community to maintain its structure and function under environmental disturbance. CS indicated the stability of microbial community composition and its resistance to fluctuation. EA represented the activity level of soil enzymes involved in nutrient transformation and microbial metabolic processes. These indicators were used to characterize the ecological functional status of pre-sowing soil and its capacity to respond to FSWI-induced environmental changes.

For each soil sample stored at minus 80 °C, approximately 0.25 g of soil was weighed, and total genomic DNA was extracted using the E.Z.N.A. Soil DNA Kit (Omega Bio Tek, USA) in strict accordance with the manufacturer’s instructions. The concentration, purity as indicated by the A260/A280 ratio, and integrity of the extracted DNA were assessed using a NanoDrop 2000 ultramicro spectrophotometer (Thermo Fisher Scientific, USA) and 1% agarose gel electrophoresis. All qualified DNA samples were uniformly diluted to a final concentration of 10 ng/μL and stored at minus 20 °C for subsequent PCR amplification.

To characterize microbial properties, the 16S rRNA gene and the ITS region were amplified. The V3 to V4 hypervariable region of the bacterial 16S rRNA gene was amplified using the primer pair 338F (5′ ACTCCTACGGGAGGCAGCAG 3′) and 806R (5′ GGACTACHVGGGTWTCTAAT 3′), each carrying a sample specific barcode. The fungal ITS1 region was amplified using the primer pair ITS1F (5′ CTTGGTCATTTAGAGGAAGTAA 3′) and ITS2R (5′ GCTGCGTTCTTCATCGATGC 3′). Each PCR reaction was performed in a total volume of 25 μL, containing 12.5 μL of 2× Phusion High Fidelity PCR Master Mix with GC Buffer, 0.5 μM of each forward and reverse primer, and 10 ng of template DNA. The amplification program consisted of an initial denaturation at 98 °C for 1 min, followed by 30 cycles of 98 °C for 10 s, 60 °C for 30 s and 72 °C for 30 s, and a final extension at 72 °C for 5 min. Three independent PCR replicates were performed for each sample to minimize random amplification bias. The three PCR products from the same sample were pooled in equal amounts and purified using the AxyPrep DNA Gel Extraction Kit (Axygen Biosciences, USA). The purified amplicons were then accurately quantified on a fluorometer using the Quant iT PicoGreen dsDNA Assay Kit (Thermo Fisher Scientific, USA). Subsequently, amplicons from all samples were pooled at equimolar concentrations, and sequencing libraries were constructed using the DNBSEQ T7 RS Kit (FCL PE150) v2.0. Paired end sequencing with a read length of 150 bp was finally performed on the DNBSEQ T7 platform (BGI Tech Solutions, China).

The raw sequencing data generated from the platform were first demultiplexed according to their barcodes. The demultiplexed reads were then quality controlled using Fastp (v0.20.0), including removal of adapter sequences, low quality reads with default parameters of Phred score below 20, and excessively short sequences, to obtain high quality clean reads. Operational taxonomic unit clustering was subsequently performed on the clean reads at a 97% similarity threshold using the UPARSE algorithm implemented in USEARCH v10. Before clustering, all sequences were screened for chimeras. Taxonomic annotation of representative OTU sequences was carried out using the RDP Classifier (v2.2) based on a Bayesian algorithm, with the Silva database (Release 138) as the reference for 16S rRNA gene sequences and a confidence threshold of 70%. Tagged sequences were then mapped to the representative OTU sequences to obtain abundance information for both OTUs and their taxonomic annotations.

#### 2.3.4 Crop growth and grain quality

Measurements of sunflower growth and yield traits at maturity were conducted in each plot by randomly selecting representative plants along a diagonal transect or using a five point sampling method, while avoiding border rows to minimize edge effects. Plant height was measured with a tape measure as the vertical distance from the soil surface to the top of the capitulum. Leaf area length and width were measured on fully expanded leaves with a tape measure, and leaf area index was then calculated based on sunflower planting density. Capitulum diameter was measured using a vernier caliper or ruler as the maximum diameter of the capitulum and the diameter perpendicular to it, and the mean of the two values was taken as the final capitulum diameter. For plant dry weight, the aboveground parts were harvested at a uniform height, returned to the laboratory, pre dried at 105 °C for 30 min, and then oven dried at 65 °C to constant weight before weighing. Thousand seed weight was determined by randomly sampling seeds from the threshed and homogenized grain of each plot, either by counting and weighing 1,000 seeds directly or by weighing 100 seeds in ten replicates and converting the result to thousand seed weight. Yield was determined from the actual harvested seed mass in each plot. After impurities were removed, seed moisture content was measured and standardized to the prescribed moisture level, and yield was then converted to kg ha^−1^ according to plot area.

Sunflower seed quality traits were determined after harvest by randomly selecting representative seeds from the threshed and homogenized sample of each plot. After impurities were removed, the seeds were ground and sieved, and then uniformly dried at 60 °C to constant weight or adjusted to a common moisture basis according to a standard moisture determination method to ensure comparability among treatments. Seed oil content and crude fat content were determined by Soxhlet extraction, using petroleum ether or n hexane as the solvent. Samples were continuously extracted under constant temperature reflux for the prescribed duration, after which the solvent was evaporated and the extracted fat was weighed. Results were expressed as a percentage on a dry weight basis. Oleic acid content was determined by gas chromatography. The extracted oil was first methylated to produce fatty acid methyl esters, which were then separated and detected by GC and quantified using an internal standard method. Oleic acid content was expressed as a percentage of total fatty acid composition. Protein content was determined by the Kjeldahl nitrogen method or elemental analysis. Total nitrogen content was measured and multiplied by a conversion factor to obtain crude protein content, and the result was expressed as a percentage on a dry weight basis.

### 2.4 Data analysis

#### 2.4.1 Crop indicator evaluation method

To comprehensively characterize the overall effects of FSWI on crop growth and seed quality, this study combined standardization with principal component analysis to construct a comprehensive growth index (GI) and a comprehensive quality index (QI), thereby enabling unified comparison and integrated evaluation across treatments.

First, the sets of growth indicators and quality indicators were standardized separately using the z score method, as shown in Equation 3, to eliminate dimensional differences and ensure that all variables contributed to the dimensionality reduction analysis on a common scale:

(3) $$Z_{ij}=\frac{x_{ij}-{\bar{x}}_{j}}{s_{j}}$$

where *x_ij_* is the observed value of the *j*th indicator for the *i*th sample, ${\bar{x}}_{j}$ and *s_j_* are the mean and standard deviation of that indicator, respectively, and *Z_ij_* is the standardized dimensionless value.

Subsequently, principal component analysis was performed on the standardized matrix to obtain a set of principal component scores, denoted as *PC_k_*. Each principal component was constructed as a linear combination of the original indicators using Equation 4:

(4) $$PC_{ik}=\sum_{j=1}^{p}a_{jk}Z_{ij}$$

Where *a_jk_* is the loading coefficient of the *j*th indicator on the *k*th principal component, *p* is the number of indicators, and *PC_ik_* is the score of sample *i* on principal component *k*.

Principal components with the highest explanatory power were retained to construct and visualize the composite indices. The composite index was calculated as a weighted sum based on the explained variance of the retained principal components using Equation 5:

(5) $$GI_{i}=\sum_{k=1}^{m}w_{k}\;PC_{ik},QI_{i}=\sum_{k=1}^{m}w_{k}\;PC_{ik}$$

where the weight *w_k_* was determined by the variance contribution rate of each principal component using Equation 6:

(6) $$w_{k}=\frac{\lambda_{k}}{\sum_{k=1}^{m}\lambda_{k}}$$

where λ*_k_* is the eigenvalue, or variance contribution, of the *k*th principal component. To ensure that the directions of the indices had clear agronomic meaning, the index orientations were further calibrated against yield and oil content, such that a higher GI corresponded to an overall improvement in growth and yield, whereas a higher QI indicated an overall enhancement in seed quality. Specifically, the direction of each composite index was examined according to its correlation with yield or oil content, and when a negative correlation was observed, the sign of the entire index was reversed using Equations 7 and 8:

(7) $$GI_{i}^{*}=\text{sign}\left(\text{corr}\left(GI,Yield\right)\right)\cdot GI_{i}$$

(8) $$QI_{i}^{*}=\text{sign}\left(\text{corr}\left(QI,Oil\right)\right)\cdot QI_{i}$$

thereby ensuring that GI and QI remained consistent and comparable in their interpretation across different treatments.

#### 2.4.2 XGBoost-SHAP analysis method

To overcome the limited interpretability arising from the black box nature of the XGBoost model, this study introduced the game theory based SHAP method, namely Shapley Additive Explanations, to interpret model predictions. SHAP expresses the model output for any sample *x* as an additive decomposition consisting of a baseline value plus the sum of feature contributions, as shown in Equation 9:

(9) $$\hat{y}\left(x\right)=\phi_{0}+\sum_{i=1}^{M}\phi_{i}\left(x\right)$$

where $\hat{y}\left(x\right)$ is the model prediction for sample *x* (in this study, the predicted value of crop yield *K*), and *M* is the number of features. *ϕ*_0_ is the baseline value, which is usually defined as the expected model output using Equation 10:

(10) $$\phi_{0}=E_{X}\left[f\left(X\right)\right]$$

Accordingly, the deviation of the prediction from the baseline can be decomposed into the sum of the contributions of all input variables using Equation 11:

(11) $$f\left(x\right)-E\left[f\left(X\right)\right]=\sum_{i=1}^{M}\phi_{i}\left(x\right)$$

where *ϕ_i_*(*x*) is the SHAP value of the *i*th feature for sample *x*, representing the marginal contribution of that feature to the prediction. Its theoretical definition is the weighted average of the Shapley value, as shown in Equation 12:

(12) $$\phi_{i}\left(x\right)=\sum \frac{\left(\left|S\right|!\left(M-\left|S\right|-1\right)!\right)}{M!}\left[f_{S\cup \left\{i\right\}}\left(x\right)-f_{S}\left(x\right)\right]$$

where *F* = {1,2,…, *M*} denotes the full set of features, *S* is any subset of features that does not include feature *i*, and |*S*| is the size of that subset. *f_s_*(*x*) denotes the model output based only on the feature subset *S*, whereas $f_{S\cup \left\{i\right\}}\left(x\right)-f_{S}\left(x\right)$ represents the marginal gain contributed by feature *i* given subset *S*. The weighting term $\frac{|S|!\left(M-|S|-1\right)!}{M!}$ ensures a fair allocation of contributions across all possible feature combinations. Accordingly, the sign of *ϕ_i_*(*x*) indicates the direction of contribution: *ϕ_i_*(*x*) > 0 means that feature *i* drives the prediction above the baseline, whereas *ϕ_i_*(*x*)< 0 means that it suppresses the prediction. The magnitude |*ϕ_i_*(*x*)| reflects the strength of the effect.

At the global level, to characterize the importance of each variable to the overall model output, this study used the mean absolute SHAP value across all samples as the measure of feature importance using Equation 13:

(13) $$I_{i}=\frac{1}{N}\sum_{j=1}^{N}|\phi_{i}\left(x^{\left(j\right)}\right)|$$

where *I_i_* is the global importance of the *i*th feature, *N* is the number of samples, and *x*^(^*^j^*^)^ is the *j*th sample. At the local level, the contribution of each feature to an individual sample can be directly decomposed using Equation 14:

(14) $$\hat{y}\left(x^{\left(j\right)}\right)=\phi_{0}+\sum_{i=1}^{M}\phi_{i}\left(x^{\left(j\right)}\right)$$

thereby revealing the strength of variable effects and their differences under different sample conditions.

In this study, SHAP values were calculated in the RStudio environment using the SHAP for XGBoost package. The input features included SWC, EC, TSC, SAR, PD, PC, ST, STG, MD, Rob, CS and EA, and the target variable was crop yield. The XGBoost model was trained using 36 matched observations, corresponding to 4 FSWI treatments × 3 replicates × 3 experimental years. Each observation included 12 soil functional predictors and the corresponding crop yield; therefore, the sample size for each input variable was 36. Considering the limited sample size, sixfold cross-validation was used during model training to reduce the risk of overfitting and improve model stability.

The XGBoost-SHAP analysis was designed to identify the effects of pre-sowing soil functional indicators on yield formation. Therefore, only soil water-salt, structural, thermal and microbial indicators measured before sowing were used as input predictors. GI and QI were not included in the driver analysis because they were integrated crop response indices rather than independent soil driving factors. In particular, GI was constructed using yield as one of its component variables; including GI as a predictor of yield would introduce circular reasoning and data leakage. QI was also a post-harvest quality response index and did not represent a pre-sowing soil driver. Therefore, GI and QI were used for comprehensive treatment evaluation, whereas XGBoost-SHAP was used to identify soil functional drivers of yield.

## 3 Results

### 3.1 Response patterns of soil function

**Figure 3:**
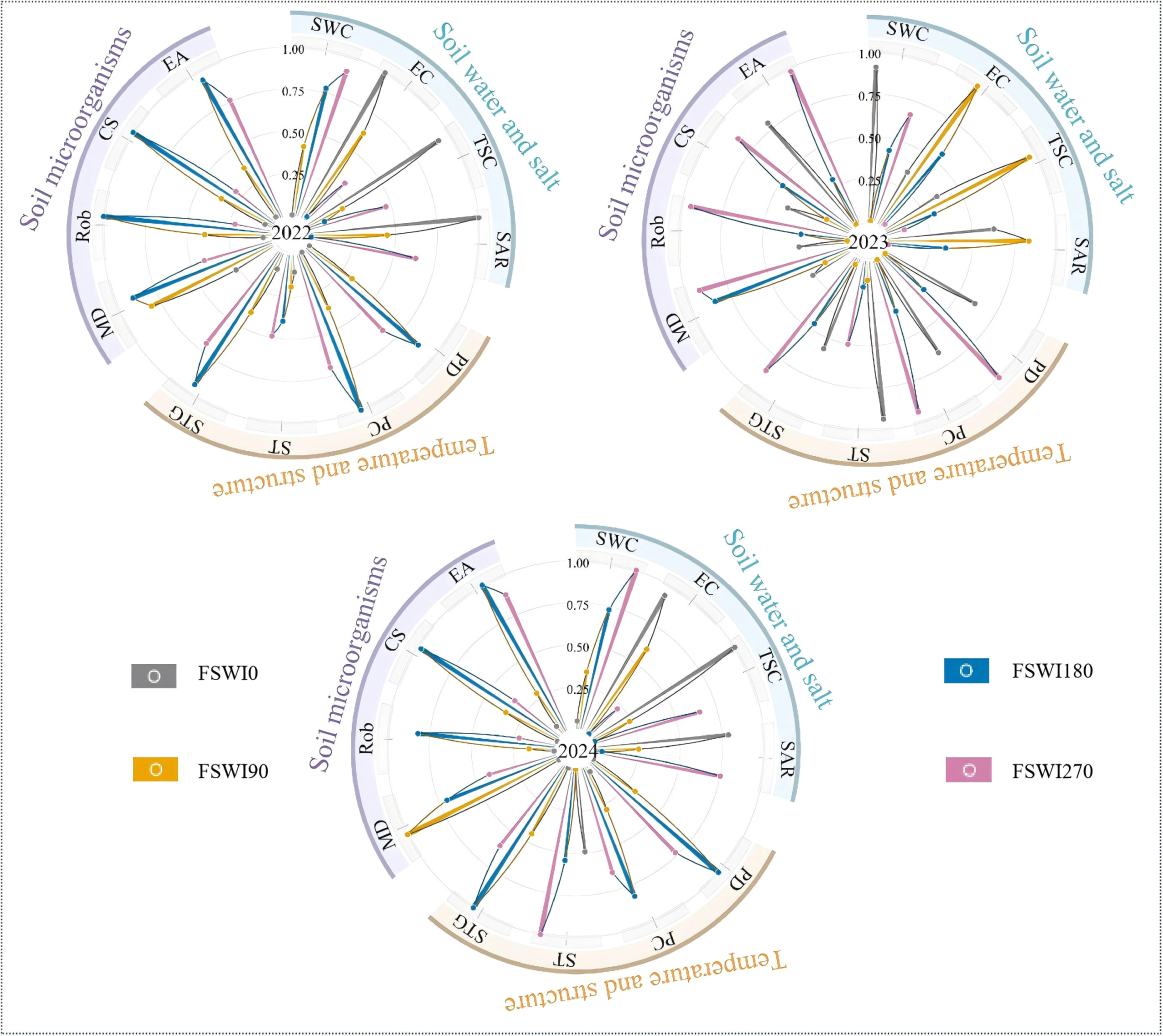
Interannual variations in soil microbial, soil water-salinity, temperature, and structural indices. Radar plots show the normalized distribution of each indicator in 2022, 2023, and 2024. Purple, blue, and orange sectors denote soil microbial, soil water-salinity, and soil temperature-structure indices, respectively. All values were normalized to 0–1, and different colored lines represent different treatments. EA, Enzyme activity; CS, Community stability; Rob, Community robustness; MD, Microbial diversity; SWC, Soil water content; EC, Electrical conductivity; TSC, Total salt content; SAR, Sodium adsorption ratio; PD, Pore diameter; PC, Pore connectivity; ST, Soil temperature; STG, Soil temperature gradient.

The standardized analysis showed that FSWI at different irrigation amounts markedly affected pre sowing soil function, and that the overall patterns were consistent from 2022 to 2024 (**Figure 3**). Among all treatments, FSWI180 consistently produced the best overall effects across the three years. The standardized values of SWC under this treatment were 0.855, 0.473 and 0.717, those of EC were 1.000, 0.454 and 1.000, those of TSC were 1.000, 0.766 and 1.000, and those of SAR were 1.000, 0.591 and 1.000. Their three year mean values reached 0.682, 0.818, 0.922 and 0.864, respectively. These results indicate that FSWI180 was able to maintain a relatively stable balance among soil water storage, salt reduction and mitigation of sodification risk under different annual conditions, making it the most effective treatment for water and salt regulation. By contrast, although FSWI90 also showed some improvement, the magnitude of its benefits was limited, with three year mean values of only 0.255, 0.244, 0.535 and 0.420 for SWC, EC, TSC and SAR, respectively. FSWI270 showed the highest mean SWC value at 0.911, indicating that a high irrigation amount enhanced soil water storage capacity. However, its mean TSC and SAR values were only 0.633 and 0.473, respectively, both clearly lower than those of FSWI180, suggesting that although excessive irrigation improved water storage, it also increased the risk of salt rebound and sodification.

In terms of soil temperature and structural function, FSWI180 also exhibited favorable characteristics. Under this treatment, PD and PC both reached 1.000 in 2022 and 2024, and were 0.314 and 0.347, respectively, in 2023. Their three year mean values were 0.771 and 0.782, both markedly higher than those under FSWI90, for which both values were 0.226. This indicates that an appropriate irrigation amount was more conducive to the formation of a more direct and better connected pore structure. Although the mean PD and PC values under FSWI270 reached 0.829 and 0.824, respectively, which were slightly higher than those under FSWI180, this advantage was mainly concentrated in 2023 and 2024. When considered together with its weaker performance in water and salt regulation and microbial indicators, this structural advantage did not translate into superior overall soil function. At the same time, the mean ST and STG values under FSWI180 were 0.449 and 0.840, respectively, indicating that this treatment not only maintained a relatively stable soil thermal environment, but also favored heat exchange during the freeze thaw period. FSWI270 had the highest mean ST value at 0.824, but this thermal advantage was insufficient to offset the risks of salt accumulation and ecological functional decline associated with excessive irrigation.

Soil microbial function showed even greater sensitivity to irrigation amount. The three year mean values of MD, Rob, CS and EA under FSWI180 were 0.871, 0.764, 0.822 and 0.768, respectively, all of which were at relatively high levels. Notably, all four indicators reached 1.000 in 2022, and Rob, CS and EA also reached 1.000 in 2024, indicating that FSWI180 substantially enhanced microbial activity and improved both the robustness and stability of community structure. By comparison, the mean MD value under FSWI90 was 0.623, suggesting that the low irrigation treatment still exerted a certain positive effect on microbial diversity. However, its mean Rob, CS and EA values were only 0.182, 0.224 and 0.206, respectively, indicating limited effects on microbial activity and community optimization. The mean EA value under FSWI270 reached 0.894, suggesting that soil enzyme activity could still be maintained at a relatively high level under high irrigation conditions. However, its mean MD, Rob and CS values were only 0.551, 0.481 and 0.538, all lower than those under FSWI180, indicating that excessive irrigation may weaken the structural integrity and stability of the microbial community.

Overall, the standardized results consistently showed that FSWI180 provided the most favorable pre-sowing soil functional background. This treatment not only promoted water storage and salt reduction, but also improved soil pore structure, enhanced microbial activity and increased community stability. Although FSWI90 exerted certain positive effects, its improvements were limited, whereas FSWI270 increased soil moisture but was accompanied by weaker salt regulation and reduced microbial functional stability. These results indicate that the advantage of FSWI180 originated from coordinated reshaping of soil water-salt, structural and microbial functions before sowing, providing a functional basis for interpreting subsequent crop growth, yield and quality responses.

### 3.2 Crop growth and seed quality

**Figure 4:**
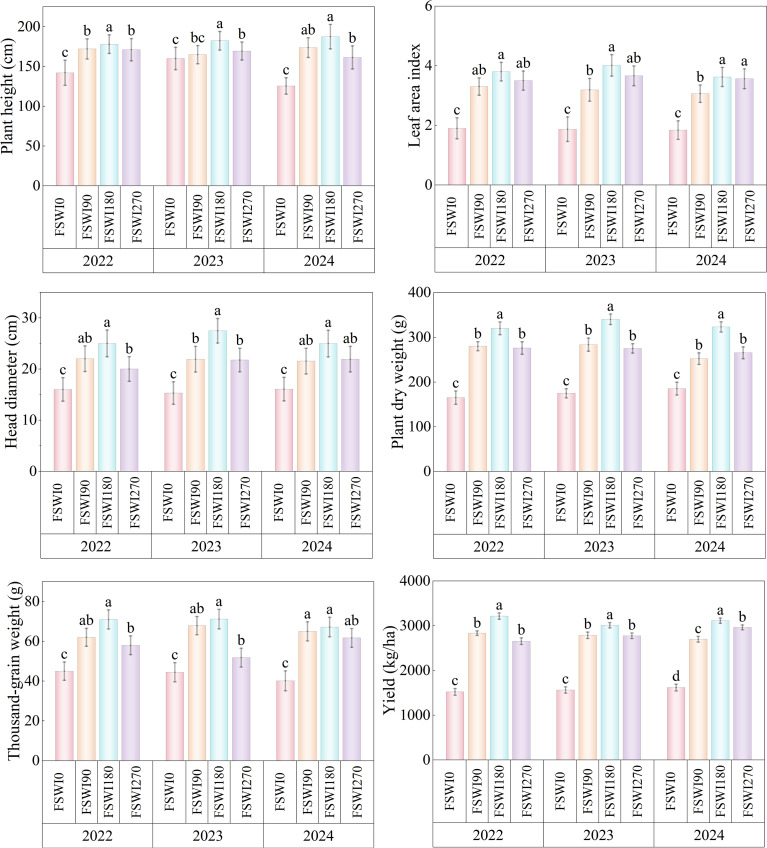
Effects of different FSWI amounts on sunflower growth traits and yield in 2022–2024. Values are presented as mean ± SD (*n* = 3). Different lowercase letters indicate significant differences among treatments within the same year.

From 2022 to 2024, FSWI significantly affected crop growth and yield, with all indicators showing a consistent nonlinear response to irrigation amount (**Figure 4**). Across years, FSWI180 consistently produced the best overall performance, followed by FSWI90, whereas FSWI270 declined relative to FSWI180 and FSWI0 remained the poorest, indicating a clear optimum rather than a monotonic benefit from greater irrigation. Treatment ranking was generally stable across years, although differences were more pronounced in 2024. For instance, plant height under FSWI0 declined to 125.45 cm in 2024, whereas FSWI180 remained at 187.40 cm, creating a difference of 61.95 cm. Leaf area index ranged from 1.84 to 4.01 overall, and under FSWI180 reached 3.80, 4.01 and 3.62 from 2022 to 2024, compared with 1.90, 1.87 and 1.84 under FSWI0. Dry matter accumulation under FSWI180 was 320.00, 340.09 and 323.14 g, consistently higher than the 165.00, 174.70 and 185.26 g recorded under FSWI0. Capitulum diameter increased from 16.00, 15.30 and 16.06 cm under FSWI0 to 25.00, 27.47 and 24.97 cm under FSWI180, while thousand seed weight increased from 45.0, 44.45 and 40.11 g to 71.0, 71.17 and 67.16 g. Final yield under FSWI180 was approximately 111%, 93% and 93% higher than under FSWI0, confirming that moderate irrigation most effectively improved crop performance.

**Figure 5:**
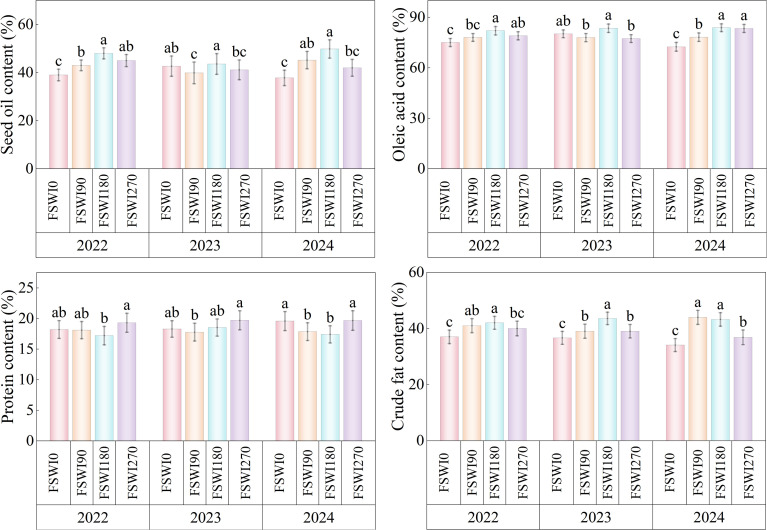
Effects of different FSWI amounts on sunflower seed quality traits in 2022–2024. Values are presented as mean ± SD (*n* = 3). Different lowercase letters indicate significant differences among treatments within the same year.

Seed quality traits showed clear nonlinear responses to FSWI intensity (**Figure 5**). Overall, moderate irrigation favored oil related quality, whereas high irrigation promoted protein accumulation, indicating a tradeoff between oil quality improvement and protein gain. Oil content was the most sensitive trait. FSWI180 consistently produced the highest values across 2022 to 2024, reaching 48.37%, 43.96% and 49.10%, representing increases of 23.4%, 3.2% and 27.2%, respectively, relative to FSWI0. Although FSWI270 also increased oil content, the effect was weaker, and in 2023 its value was only 40.85%, lower than the 42.62% recorded under FSWI0. Oleic acid content showed the same pattern, peaking under FSWI180 at 82.00%, 84.28% and 83.80%, which were 10.8%, 11.0% and 6.8% higher than FSWI0, respectively. In contrast, protein content was highest under FSWI270, reaching 19.30%, 19.71% and 19.67%, or 9.0%, 7.2% and 4.0% above FSWI0. Crude fat content was highest under FSWI180 in 2022 and 2023 at 42.00% and 43.58%, and in 2024 under FSWI90 at 43.95%, only slightly above FSWI180 at 43.22%, with both exceeding FSWI0 at 33.89%. These crop responses suggest that the benefits of FSWI180 were transmitted from pre-sowing soil improvement to aboveground growth, yield formation and seed quality, providing a basis for the subsequent integrated evaluation.

### 3.3 Comprehensive crop evaluation based on PCA

**Figure 6:**
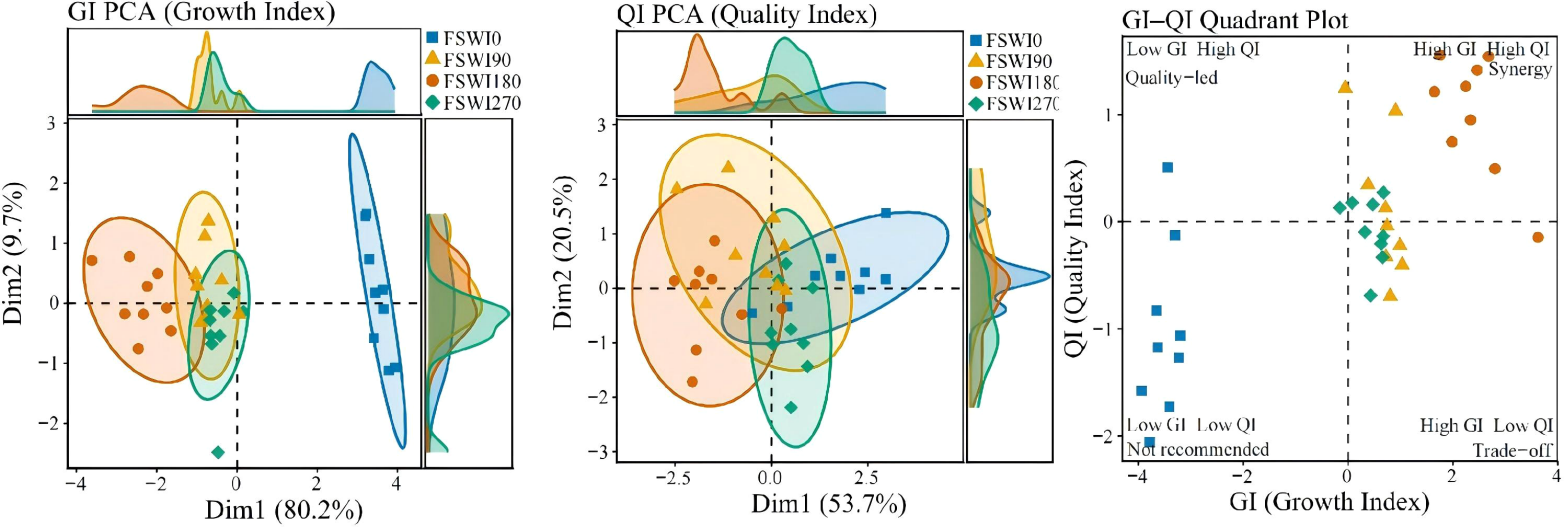
Comprehensive evaluation of sunflower growth and seed quality based on PCA. Each point represents one plot-year observation. For each FSWI treatment, nine samples were included, corresponding to three replicates across three experimental years from 2022 to 2024 (*n* = 9). Ellipses indicate the distribution of samples within each treatment in the PCA space.

To comprehensively evaluate the overall effects of FSWI on crop growth and seed quality, multiple growth traits, including plant height, leaf area index, capitulum diameter, plant dry weight, thousand seed weight and yield, together with quality traits, including oil content, oleic acid content, protein content and crude fat content, were first standardized and then analyzed by principal component analysis (**Figure 6**). Based on this, a comprehensive growth index (GI) and a comprehensive quality index (QI) were constructed. The indices were calculated as weighted sums of principal components with cumulative explained variance above 80%, and their directions were calibrated against yield and oil content so that higher GI and QI consistently represented better growth and yield performance, and better seed quality, respectively.

The suitability tests confirmed that both growth and quality datasets were appropriate for PCA. For GI, the KMO value was 0.862, and Bartlett’s sphericity test was significant (*χ²* = 239.944, *df* = 15, *P* < 0.001). For QI, the KMO value was 0.729, and Bartlett’s sphericity test was also significant (*χ²* = 26.053, *df* = 6, *P* < 0.001). For the growth traits, PC1 and PC2 explained 80.20% and 9.74% of the total variation, respectively, with a cumulative explained variance of 89.94%. For the quality traits, PC1 and PC2 explained 53.75% and 20.54% of the total variation, respectively, with a cumulative explained variance of 74.29%. These results indicate that the PCA ordination captured the major variation in growth and quality traits and provided a basis for comparing integrated crop performance among treatments. Quantitatively, FSWI180 showed the strongest overall advantage in both dimensions, with mean GI and QI values of 2.126 and 1.182, respectively, both higher than those of the other treatments. In contrast, FSWI0 had the lowest overall performance, with mean GI and QI values of −3.142 and −1.264, respectively. FSWI90 and FSWI270 remained at intermediate levels, with mean GI values of 0.584 and 0.432 and mean QI values of 0.233 and −0.151, respectively. These results indicate that moderate FSWI produced the most coordinated improvement in crop growth, yield and seed quality, whereas insufficient or excessive irrigation resulted in weaker and less stable benefits. The distribution of samples in principal component space, together with the GI–QI four-quadrant analysis, further highlighted the differences among treatments. FSWI180 samples were mainly located in the high GI and high QI quadrant, indicating coordinated improvement in growth, yield and seed quality under moderate irrigation. By contrast, FSWI0 samples were mostly distributed in the low GI and low QI quadrant, reflecting consistently poor crop performance without FSWI. FSWI90 and FSWI270 occupied transitional positions around the quadrant boundaries, suggesting that low and excessive irrigation treatments produced partial benefits but also showed evident trade-offs between growth and quality. In particular, FSWI90 showed moderate improvement in quality-related performance in some samples, whereas FSWI270 displayed greater inconsistency in the coordination between GI and QI. Taken together, the integrated evaluation based on GI and QI identified FSWI180 as the most favorable treatment under the conditions of this study. It achieved the best coordination between crop growth, yield formation and seed quality, whereas FSWI90 and FSWI270 remained transitional treatments with evident trade-offs, and FSWI0 was unsuitable for the dual goals of saline-alkaline land improvement, stable yield and high-quality production. These results provide a quantitative basis for subsequent identification of limiting factors and optimization of irrigation strategies.

### 3.4 Yield drivers and thresholds identified by XGBoost-SHAP

**Figure 7:**
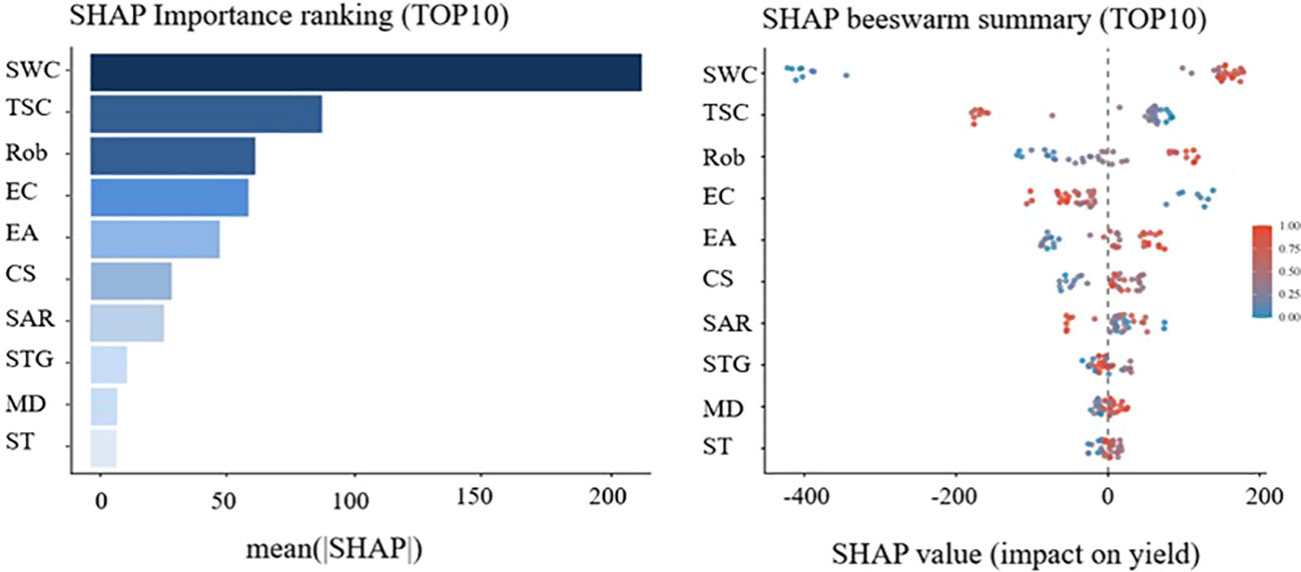
SHAP-based interpretation of yield drivers under different FSWI treatments. The left panel shows the importance ranking of the top 10 pre-sowing soil functional indicators based on mean absolute SHAP values for yield prediction. The right panel shows the SHAP beeswarm summary, where each point represents one observation and the color indicates the normalized feature value. Positive SHAP values indicate positive contributions to yield prediction, whereas negative SHAP values indicate negative contributions. SHAP importance values represent model-based feature contributions rather than traditional statistical significance levels.

To identify the dominant drivers of yield formation under FSWI, yield was set as the response variable and key soil environmental and biological indicators during the freeze thaw and ablation period were used as predictors in an XGBoost regression model, with SHAP applied to quantify variable contributions (**Figure 7**). The model was trained using 36 matched plot-year observations, with a sample size of 36 for each input variable. Sixfold cross-validation was used, and the optimal number of boosting rounds was 74, which helped reduce the risk of overfitting under the limited sample size. Therefore, the SHAP results were interpreted mainly as relative indicators of variable importance and potential nonlinear response thresholds under the experimental conditions of this study.

SHAP analysis identified SWC as the primary driver of yield, contributing 37.29% of the total importance. This indicates that effective root zone water availability was the main constraint on yield variation across irrigation treatments. The next most important factors were TSC, Rob and EC, with mean absolute SHAP values of 90.09, 64.04 and 61.43, accounting for 15.66%, 11.13% and 10.68% of total contribution, respectively. Together, these four variables explained 74.75% of the total contribution, showing that yield was governed mainly by the joint effects of water supply, salt stress and microbial resistance to disturbance.

EA, CS and SAR also contributed to yield, accounting for 8.72%, 5.48% and 5.01%, respectively, suggesting that once water and salt conditions become suitable, biological processes and sodification risk further shape yield differences. By contrast, STG, MD and ST together contributed only a small proportion and mainly acted as background modulators.

These results explain why FSWI180 performed best. Compared with FSWI0, which was mainly constrained by water deficit, and FSWI270, where higher salinity and sodification offset part of the irrigation benefit, FSWI180 maintained both adequate water supply and controllable salt stress while preserving relatively favorable microbial robustness and enzyme activity, thereby achieving more stable yield improvement.

**Figure 8:**
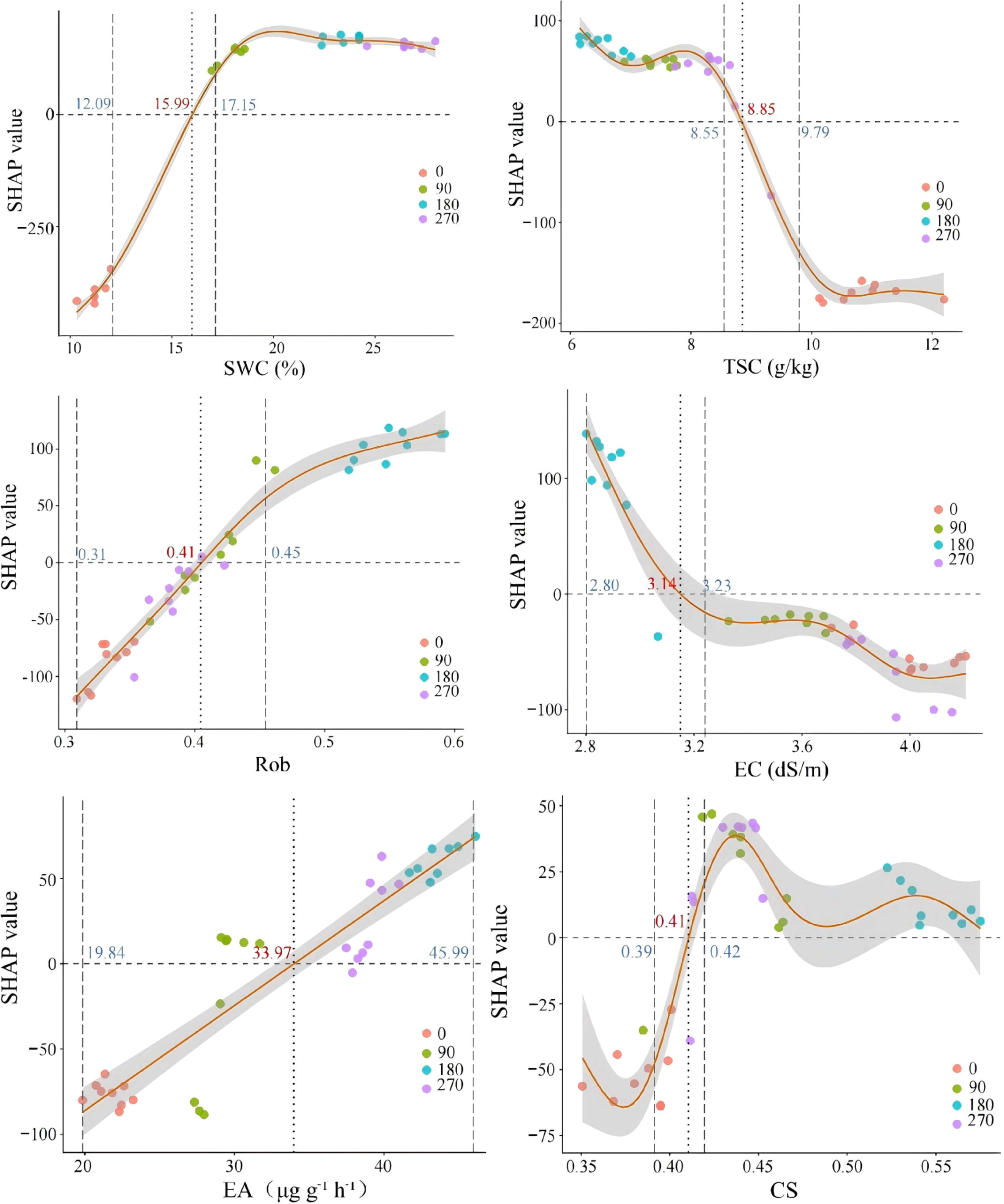
SHAP response curves for the main yield-driving variables under different FSWI treatments. soil water content (SWC), total soil salinity (TSC), microbial community robustness (Rob), electrical conductivity (EC), enzyme activity (EA) and community stability (CS). Where 0, 90, 180 and 270 represent FSWI0, FSWI90, FSWI180 and FSWI270, respectively. The orange curve represents the fitted SHAP response, and the grey band indicates the confidence interval. The horizontal dashed line indicates SHAP = 0. Red numbers indicate the variable thresholds, defined as the zero-crossing points at which the contribution of a variable to yield prediction changes direction, whereas blue-grey numbers indicate the lower and upper boundaries of the sensitive interval.

After identifying the main yield drivers, SHAP response curves were used to determine threshold values and sensitive ranges for key variables, thereby characterizing the nonlinear response of yield to soil conditions during the freeze thaw period (**Figure 8**). All major variables showed clear zero crossing points and sensitive intervals, which represent zones of rapid transition in yield contribution and thus key targets for field management.

Among all variables, SWC showed the clearest threshold pattern. Its zero crossing point was 15.99%, with a sensitive range of 12.09% to 17.15%. This indicates that yield responded most strongly to changes in root zone moisture within this interval. Below 12.09%, water deficit markedly limited yield, whereas above 17.15%, the marginal benefit of additional water declined.

Salt related variables also exhibited pronounced thresholds. TSC had a zero crossing point of 8.85 g kg^−1^ and a sensitive range of 8.55 to 9.79 g kg^−1^, indicating that around 9 g kg^−1^ was a critical salinity risk zone. EC showed a zero-crossing threshold of 3.14 dS m^−1^, with a sensitive interval of 2.80 to 3.23 dS m^−1^, suggesting that yield contribution changed rapidly as soil electrical conductivity approached approximately 3 dS m^−1^. Beyond these ranges, increasing salinity-related stress tended to suppress yield contribution.

Biological indicators mainly reflected the realization of yield potential after water and salt conditions were regulated. The zero-crossing thresholds of Rob, CS and EA were 0.41, 0.41 and 33.97 μg g^−1^ h^−1^, respectively, with corresponding sensitive intervals of 0.31 to 0.45, 0.39 to 0.42 and 19.84 to 45.99 μg g^−1^ h^−1^. These results indicate that microbial robustness, community stability and enzyme activity contributed positively to yield only after reaching certain functional levels. Overall, FSWI180 did not optimize every individual indicator, but it achieved the best coordination among the major yield-driving factors. Compared with FSWI0, which was mainly limited by water deficit, and FSWI270, where higher salinity-related stress offset part of the irrigation benefit, FSWI180 maintained a more favorable balance among soil moisture, salinity control and microbial functional support. This coordinated balance explains its higher yield and more stable integrated crop performance.

## 4 Discussion

### 4.1 Evolution of soil function and its underlying mechanisms

This study showed that FSWI markedly altered multiple pre-sowing soil functions, but its effects did not increase linearly with irrigation amount. Instead, the response reflected a balance among water replenishment, salt redistribution, alkalinity mitigation and microbial functional maintenance. Moderate FSWI was more effective in reshaping the soil environment before spring sowing, whereas excessive irrigation may weaken this coordination by increasing salinity or alkalization risks. This finding is broadly consistent with previous studies showing that an appropriate irrigation amount can increase effective root-zone moisture after freeze-thaw, promote surface salt leaching and create more favorable conditions for subsequent crop growth, whereas excessive irrigation often results in diminishing returns and may even induce salinization or alkalization risks (Li et al., 2020; Feng et al., 2021; Pan et al., 2025). These results suggest that the key role of FSWI lies not merely in increasing soil moisture, but in reshaping pre-sowing soil function through the coupled regulation of water, heat and salt dynamics during the freeze-thaw period. Consistent with earlier studies, moderate irrigation improved soil moisture while alleviating salt stress, further supporting the view that moderate salt leaching is more effective than simply increasing irrigation input in saline alkaline soil management (Liu X. et al., 2025; Lou et al., 2025). However, unlike some conventional irrigation studies, FSWI270 achieved higher soil moisture but did not lead to better overall soil function. This difference is likely related to the alternating freeze thaw conditions of the experiment. During freezing and thawing, ice water phase transitions alter soil matric potential and temperature gradients, thereby driving vertical and lateral redistribution of water and salts (Tang et al., 2021; Wang et al., 2022). Although higher irrigation may enhance short term water storage, it may also increase salt return and alkalization risks, weakening the overall benefit. Moreover, improved pore connectivity does not necessarily imply improved soil function, because when EC and SAR remain high, soil aggregate stability, ionic conditions and microbial habitat may still be constrained (Wang et al., 2025; Obregon et al., 2025). This suggests that under freeze thaw saline alkaline conditions, water and salt processes, structural evolution and microbial responses are governed by stronger thresholds and tighter coupling, which also explains why the present results do not fully align with some findings obtained under non freeze thaw conditions.

Mechanistically, the superiority of FSWI180 appears to arise from coordinated matching among water supply, salt migration, structural optimization and biological recovery. Moderate irrigation increased root zone water availability after freeze thaw, promoted downward salt migration during thaw infiltration, alleviated surface salt accumulation and ionic stress, and favored pore reorganization and heat exchange, thereby improving aeration and connectivity. More importantly, lower salinity and sodification pressure provided a more stable basis for microbial activity, community robustness and enzyme mediated processes, enhancing soil functional recovery (Liu et al., 2021; Jiang et al., 2024).

A major strength of this study is that it systematically revealed the nonlinear response of pre sowing soil multifunctionality under FSWI from four dimensions, namely water and salt status, structure, thermal environment and microbial processes. Compared with previous studies focusing mainly on individual water and salt indicators, this work provides a more complete picture of coordinated soil functional change. Overall, the findings indicate that the key to winter saline irrigation in saline alkaline land is not to maximize irrigation amount, but to achieve coordinated optimization of water retention, salt control, alkalinity mitigation and ecological stability through moderate irrigation. This not only deepens understanding of freeze thaw soil processes in cold arid saline alkaline regions, but also provides a more targeted basis for saline water utilization and saline alkaline land improvement.

### 4.2 Mechanisms underlying the coordinated improvement of crop growth and quality

From the overall response pattern, FSWI showed a clear stage-dependent transmission effect, whereby changes in the pre-sowing soil environment were subsequently reflected in crop growth, yield formation and seed quality. Appropriate FSWI likely promoted canopy development, dry matter accumulation and grain filling by improving root-zone water availability and alleviating salt stress, whereas excessive irrigation did not provide sustained benefits because salinity redistribution and reduced resource use efficiency may offset part of the irrigation effect (Gao et al., 2024; Lu et al., 2025). This interpretation is also supported by studies on saline irrigation and sunflower salt tolerance, which have shown that increasing salinity can restrict plant height, biomass, capitulum development and seed weight, ultimately suppressing yield formation (Han et al., 2022; Céccoli et al., 2022).A notable finding of this study is that although FSWI270 maintained relatively high soil moisture, its yield and several quality traits did not exceed those of FSWI180. This indicates that crop response depends not on water sufficiency alone, but on the balance between water supply benefits and salt stress risks. For sunflower, moderate irrigation is more favorable for canopy expansion, photosynthetic assimilation and grain filling, thereby increasing thousand seed weight and final yield, whereas excessive irrigation may offset these gains through salt return, altered aeration and fluctuations in the ionic environment (Wu et al., 2023; Zhang et al., 2024b). Previous studies have likewise emphasized that optimal irrigation in saline alkaline land should maintain root zone water and salt conditions within a range favorable for dry matter allocation and yield formation, rather than simply maximizing irrigation amount (Wang J. et al., 2025; He et al., 2025).

The quality response further showed that moderate irrigation mainly improved oil related traits, whereas higher irrigation tended to favor protein accumulation, suggesting distinct response pathways among quality components. Previous studies have shown that salinity and irrigation influence not only total yield, but also oil accumulation and fatty acid composition, indicating that high yield and high quality do not necessarily coincide and instead require more precise water and salt regulation (Ebrahimian et al., 2019; He et al., 2025). In the PCA results, FSWI180 achieved both high GI and high QI, indicating that its advantage lay not in maximizing a single trait, but in better coordinating growth, yield and quality. Overall, these findings suggest that management of FSWI in saline alkaline land should shift from yield maximization alone to coordinated optimization of yield and quality.

### 4.3 Key drivers and threshold boundaries of yield regulation

The significance of this study lies in establishing an interpretable framework to explain how soil properties and biological processes jointly regulate yield divergence under different FSWI treatments. The XGBoost-SHAP results indicate that yield was not determined by irrigation amount alone, but by the balance among root-zone water availability, salt stress intensity and soil ecological functioning. Specifically, soil moisture determined whether irrigation could be converted into effective crop water use, salinity-related indicators defined the main constraint boundaries, and microbial robustness, community stability and enzyme activity provided biological support for realizing yield potential. This interpretation is consistent with recent studies in saline-alkali agriculture and soil ecology, which suggest that rational irrigation should simultaneously improve soil water-salt status while maintaining favorable root-zone ecological processes, rather than simply increasing water supply (Du et al., 2023; Gong et al., 2025). Therefore, the XGBoost-SHAP framework provided a useful basis for capturing nonlinear relationships and clarifying the direction of variable contributions. Compared with conventional regression or single factor analysis, this study further identified threshold positions and sensitive intervals for key variables, advancing interpretation from variable ranking to process boundary definition. Soil water content showed a clear effective response interval, indicating that irrigation can be stably converted into yield gains only when root zone moisture remains within an appropriate range, whereas values below or above this range reduce marginal returns. Likewise, total salinity, EC and SAR exhibited distinct risk thresholds, showing that the effects of salinity and sodification on yield intensify rapidly near critical ranges rather than accumulating gradually. This threshold behavior agrees with previous studies showing that crop responses to soil moisture and salinity are typically nonlinear and segmented (Wang et al., 2023; Mahajan et al., 2025). More importantly, robustness, stability and enzyme activity also showed identifiable threshold intervals, indicating that once water and salt conditions are brought into suitable ranges, microbial processes become important regulators of further yield differentiation. In other words, water and salt factors define the basic boundaries of yield formation, whereas biological processes regulate the extent to which yield potential is realized within those boundaries.

From this perspective, the superiority of FSWI180 over FSWI0 and FSWI270 does not arise because a single indicator reaches its maximum, but because this treatment is more likely to satisfy three conditions simultaneously: root zone moisture remains within the effective response range, salinity and sodification stay within controllable limits, and microbial community status together with enzyme mediated processes remain favorable. The novelty of this study lies in translating the mechanism underlying the optimal treatment into coordinated matching among a water interval, salinity boundaries and biological functional thresholds, thereby providing a more operational basis for optimizing FSWI. It should be noted, however, that the XGBoost-SHAP analysis was based on 36 matched plot-year observations. Therefore, the identified thresholds mainly represent potential statistical transition ranges under the specific soil, climatic and management conditions of this study, rather than universal management thresholds. Their stability across different salinity types, groundwater depths, climatic years and larger spatial scales still requires further validation with larger datasets. Even so, this work provides a basis for shifting irrigation management in cold arid saline alkaline regions from empirical judgment to quantitative early warning, and suggests a new direction for integrating interpretable machine learning with long term field experiments, process based models and multiscale monitoring.

## 5 Conclusions

This study demonstrated that the effects of FSWI on saline-alkali soil function and sunflower performance were nonlinear, and that greater irrigation amount did not necessarily lead to greater benefits. Among the tested treatments, FSWI180 showed the most favorable overall performance by coordinating pre-sowing soil water availability, salinity regulation, soil structural conditions and microbial functional status during the freeze-thaw period. These improvements were subsequently reflected in better sunflower growth, higher yield and improved seed quality under uniform agronomic management. In contrast, FSWI90 provided limited benefits because of insufficient water supplementation, whereas FSWI270 increased soil moisture but weakened the overall improvement due to salinity-related risks and less stable microbial responses. The integrated PCA and XGBoost-SHAP analyses further showed that yield formation was mainly controlled by the combined effects of SWC, TSC, EC and microbial community robustness, rather than by irrigation amount alone or any single soil factor. Although FSWI180 did not optimize every individual indicator, it achieved the best balance among the key yield-driving factors. These findings suggest that FSWI management in cold arid saline-alkali regions should focus on coordinated regulation of water storage, salt control and microbial function. Under the conditions of this study, 180 mm can serve as a practical reference amount for improving soil conditions and supporting stable, high-quality sunflower production.

## Data Availability

The datasets presented in this study are deposited in the Figshare repository, DOI: https://doi.org/10.6084/m9.figshare.32494272.
